# Planned Pregnancy in Kidney Transplantation. A Calculated Risk

**DOI:** 10.3390/jpm11100956

**Published:** 2021-09-26

**Authors:** Claudio Ponticelli, Barbara Zaina, Gabriella Moroni

**Affiliations:** 1 Nephrology, Ospedale Maggiore Policlinico, 20122 Milan, Italy; 2 Department of Obstetrics and Gynecology, IRCCS Ca’ Granda Ospedale Maggiore Policlinico, 20122 Milan, Italy; barbara.zaina@policlinico.mi.it; 3 Department of Biomedical Sciences, IRCCS Humanitas Research Hospital, Humanitas University, 20122 Milan, Italy; gabriella.moroni@hunimed.eu

**Keywords:** posttransplant pregnancy, fetotoxicity, eclampsia, immunosuppression

## Abstract

Pregnancy is not contraindicated in kidney transplant women but entails risks of maternal and fetal complications. Three main conditions can influence the outcome of pregnancy in transplant women: preconception counseling, maternal medical management, and correct use of drugs to prevent fetal toxicity. Preconception counseling is needed to prevent the risks of an unplanned untimely pregnancy. Pregnancy should be planned ≥2 years after transplantation. The candidate for pregnancy should have normal blood pressure, stable serum creatinine <1.5 mg/dL, and proteinuria <500 mg/24 h. Maternal medical management is critical for early detection and treatment of complications such as hypertension, preeclampsia, thrombotic microangiopathy, graft dysfunction, gestational diabetes, and infection. These adverse outcomes are strongly related to the degree of kidney dysfunction. A major issue is represented by the potential fetotoxicity of drugs. Moderate doses of glucocorticoids, azathioprine, and mTOR inhibitors are relatively safe. Calcineurin inhibitors (CNIs) are not associated with teratogenicity but may increase the risk of low birth weight. Rituximab and eculizumab should be used in pregnancy only if the benefits outweigh the risk for the fetus. Renin–angiotensin system inhibitors, mycophenolate, bortezomib, and cyclophosphamide can lead to fetal toxicity and should not be prescribed to pregnant women.

## 1. Introduction

In most women with successful kidney transplantation, fertility is spontaneously restored within a few months, resulting in normal ovulatory cycles and regular menstruation [1,2,3]. This offers a chance for transplanted women to have children. Pregnancy can be a reasonable option for women with a good function of the kidney transplant. However, pregnancy in transplanted patients remains high risk [4]. A meta-analysis and systematic review of 6712 pregnancies in 4174 kidney transplant recipients reported that the live-birth rate was 72.9%. The other pregnancy outcomes were complicated by cesarean section (62.6%), preterm delivery (43.1%), pregnancy-induced hypertension (24.1%), preeclampsia (21.5%), miscarriages (15.4%), induced abortions (12.4%), gestational diabetes (5.7%), stillbirths (5.1%), and ectopic pregnancies (2.4%) [5].

In this article, the main potential issues of pregnancy in kidney transplantation will be discussed. Particular attention will be focused on three main topics: the counseling on contraception, the maternal medical management during pregnancy, and the choice of immunosuppressive drugs to prevent fetal toxicity.

## 2. Planning Posttransplant Pregnancy

Pregnancy leads to physiologic changes in kidney and systemic hemodynamics that cause important alterations in acid–base, electrolyte, and kidney function. Glomerular hyperfiltration occurs during pregnancy. An increase in 24 h creatinine clearance by 25% occurs in the first 4 weeks after the last menstrual period and by 45% at 9 weeks. During the third trimester, there is some decrease in creatinine clearance with a small increase in the first days of puerperium. An increase in plasma uric acid concentration in pregnancy may predict the development of preeclampsia. Proteinuria may develop in almost 40% of patients near term but disappears after delivery. Transient diabetes insipidus may occur late in pregnancy. It disappears with puerperium but may be responsible for hypovolemia and electrolyte unbalance [6]. Understanding these changes is essential when evaluating pregnant women with kidney transplantation, outlining that transplanted pregnant women should be closely monitored and followed by a team of expert nephrologists and gynecologists. 

The potential issues of pregnancy should be discussed, preferably prior to transplantation, by women and their partners with a multidisciplinary team of expert nephrologists and obstetricians. Such issues include preconception counseling, knowledge of factors that may affect pregnancy success, the timing of pregnancy, and the possible impact of medical management on maternal and fetal outcomes [7,8].

Early counseling on contraception is important to reduce the risk of unplanned pregnancies, improve pregnancy outcomes, and reduce maternal complications. Hormonal methods may be safe in women with stable graft function but are not recommended in women with hypertension or risk of thrombosis. Indeed, arterial hypertension during pregnancy is associated with increased arterial thrombotic risk [9]. All combined oral contraceptives investigated in an analysis were associated with an increased risk of venous thrombosis; the effect size depended both on the progestogen used and the dose of ethinylestradiol [10]. Intrauterine devices, oral contraceptives, and subdermal implants have been shown to have great efficacy as well as low systemic drug absorption, allowing clinicians to provide a vast range of options to transplant women in their childbearing years [11,12,13]. Counseling should be repeated after transplantation [14]. Emergency contraception with high dose progestins or intrauterine devices is not contraindicated, but it should be avoided whenever possible, even if far preferable to abortion [15].

Several factors may affect pregnancy outcomes, including rejection, poor graft function, nephrotic syndrome, hypertension, obesity, diabetes, and infections. According to the National Transplantation Pregnancy Registry (NTPR), the ideal candidate for posttransplant pregnancy should not have had rejection in the last year, should have adequate and stable kidney graft function (serum creatinine < 1.5 mg/dl and protein exception < 500 mg/24 h urine protein excretion), normal blood pressure (with or without therapy), and stable immunosuppression [8]. She should not suffer from comorbid factors that may worsen pregnancy outcomes, such as chronic allograft dysfunction, cardiopulmonary diseases, severe hypertension, diabetes mellitus, nephrotic proteinuria, morbid obesity, maternal infection with hepatitis B or C, and severe liver failure.

The timing of conception may depend on the characteristics of the patient. The NTPR suggested that a patient can safely proceed with the pregnancy, independently of the date of transplant, if graft function is optimal [8]. However, it can be unsafe to plan conception early after transplantation. The peritransplant period is a time of the highest use of potentially fetotoxic or teratogenic medications and a time when optimization of immunosuppression is essential to prevent rejection [16]. A retrospective study of 729 pregnancies revealed an increased risk of death-censored graft loss during the first and second year after transplantation (hazard ratio 1.25 and 1.26, respectively), while pregnancy in the third year was no longer associated with an increased risk of graft failure [17]. Thus, it is safer to plan pregnancy at least 2 years after transplantation [18,19,20].

The patient should be vaccinated before pregnancy. Most live attenuated vaccines are contraindicated after transplantation. Thus, women who are not immune to rubella should receive the rubella vaccine before transplantation. However, there are emerging safety profile and efficacy data to support the use of specific live attenuated vaccines. In the posttransplant setting, inactivated vaccines can be administered starting at 3 months posttransplant, except for influenza, which can be given as early as one month. Close contacts of transplant patients can receive most routine live vaccines, including pneumococcal, influenza, hepatitis B, human papilloma virus, and meningococcal vaccines. In general, vaccines are recommended more than two weeks prior to transplantation, or starting at 1-6 months after transplantation [21]. Of particular concern is coronavirus disease 2019 (COVID-19) which is associated with a high rate of hospitalization and admission to intensive care units in kidney transplant recipients [22,23,24,25]. Since persons on immunosuppressive medications are at increased risk for severe COVID-19, it is recommended that those people may receive a COVID-19 vaccine along with counseling that the vaccine safety and efficacy profiles are unknown, and there is a potential for reduced immune responses [26].

## 3. Maternal Medical Management and Outcomes during Pregnancy

The aims of the treatment are to maintain alloimmunity quiescence during and after pregnancy and to improve the outcome of the fetus. Reaching these goals largely depends on the conditions of the patient at conception. Besides the recommendations reported above, pregnancy should be avoided in women with acute rejection in the last 12 months before conception and in patients with impaired graft function. In pregnant women with various stages of CKD, the risk of adverse outcome increased from 34% to 90% from stage 1 to 4 [27]. In many cases, prematurity and low birth weight are caused by obstetric management to favor early delivery in patients with severe CKD or preeclampsia. In these cases, it is difficult to balance the interest of the pregnant woman and her fetus. Some authors have proposed that maternal autonomy should be the dominant concern in decision making, whereas others have established the fetus as a patient who should be treated according to the principles of beneficence.

The most frequent maternal risks include infection, hypertension, preeclampsia, gestational diabetes, and acute rejection (Table 1).

### 3.1. Infection

The placental immune response and its tropism for specific viruses and pathogens affect the outcome of the pregnant woman’s susceptibility to and severity of certain infectious diseases [28]. Prophylaxis and treatment of bacterial and viral infections are important in pregnant transplant recipients. These patients are at increased risk of infections, especially bacterial urinary tract infections and acute pyelonephritis of the graft. Urine cultures should be performed monthly and all asymptomatic infections should be treated. Whether urinary tract infection impacts on graft and patient survival is still uncertain [29]. A meta-analysis in nontransplanted pregnant women reported that urinary tract infection during pregnancy increases the risk of preeclampsia [30]. Antibiotic prophylaxis for all surgical procedures is recommended. The risk of cytomegalovirus (CMV) infection during pregnancy is low in transplant women, since pregnancy is usually planned 1–2 years after transplantation. However, monitoring of CMV infections is recommended, because congenital CMV is the main nongenetic cause of congenital sensorineural hearing loss and neurological damage [31].

### 3.2. Hypertension

Pregnancy hypertension is defined as systolic blood pressure ≥ 140 mm Hg or diastolic blood pressure ≥ 90 mm Hg. About 70–90% of kidney transplant recipients have either arterial hypertension or require antihypertensive therapy [32,33]. Posttransplant hypertension is an independent risk factor for pregnancy complications [34,35]. There is no definite indication for treatment of mild–moderate hypertension in pregnancy. However, when mean arterial pressure exceeds 140 mmHg (equivalent to 180), there is a significant risk of maternal cerebral vascular damage. Oral labetalol and methyldopa are most used, and second-line agents include clonidine and calcium channel blockers [36,37]. Both angiotensin-converting enzyme inhibitors (ACE-Is) and angiotensin receptor blockers (ARBs) are contraindicated in pregnancy because single-center reports and meta-analyses revealed a significant association between overall congenital malformations and first trimester exposure to ACEI/ARBs. Cardiovascular malformations, miscarriage, and stillbirth also had a significant relation with ACEI/ARB exposure [38,39].

### 3.3. Preeclampsia

In transplanted women, pregnancies remain high risk with an increased prevalence of preeclampsia. A Norwegian retrospective cohort study based on 1,272,000 deliveries reported an adjusted incidence rate ratio of 6.06 [40]. Preeclampsia is caused by placental and maternal vascular dysfunction and should not be confused with cases of de novo hypertension or proteinuria [41]. Today, preeclampsia is considered a systemic disease with widespread endothelial damage and the potential to affect future cardiovascular diseases rather than a self-limited occurrence. Underlying mechanism(s) include heme oxygenase and hydrogen sulfide pathways, autoantibodies, misfolded proteins, nitric oxide, oxidative stress, extracellular vesicles containing miRNA, and other important nucleotides [42,43]. These mechanisms can result in abnormal placentation, inadequate spiral artery remodeling, deficiency in trophoblast invasion, reduced maternal blood flow to the placenta, and a release of signals and/or inflammatory mediators into maternal circulation, triggering the systemic manifestations of preeclampsia. The risk of preeclampsia in pregnant transplant women might be related to poor graft function, hypertension, and immunosuppressive therapy. In pregnant women, glucocorticoids may cause adverse events that are mainly related to their dosage and duration, but whether the use of glucocorticoids can increase the risk of preeclampsia is still uncertain. In pregnant patients with autoimmune disease taking glucocorticoids, confounding factors, including the dosage of prednisone and the nature and severity of the underlying autoimmune disease, were not detailed [44,45,46]. The only study that adjusted for disease and disease severity did not find evidence of an association between prednisone use and preeclampsia [47]. Hypertension and preeclampsia have been reported to occur at higher incidence in transplanted women taking calcineurin inhibitors [48,49]. A systematic review and meta-analysis could not find any difference in the rate of preeclampsia between transplant pregnant women taking cyclosporine or tacrolimus [50].

Signs and symptoms of preeclampsia include hypertension, proteinuria, diffuse edema, belly pain in the upper right side, gastric troubles, and vision changes. In a study from Norway, transplant women with preeclampsia had significantly higher postpartum serum creatinine levels, higher risks of preterm delivery, cesarean delivery, and small for gestational age infants. In the final multivariate model, chronic hypertension, previous preeclampsia, and elevated serum creatinine at the start of pregnancy were prognostic factors for preeclampsia [51]. In the most severe forms, preeclampsia can lead to eclampsia, defined as the new onset of generalized tonic–clonic seizures. Eclampsia is a life-threatening condition for the mother and fetus. The early treatment of acute hypertension, use of magnesium sulfate, and early hospitalization may prevent the development of eclampsia, but the only effective treatment is delivery. Although preeclampsia has not been found to have a deleterious effect on kidney graft function in transplant women, it can cause premature delivery [52,53]. The drug of choice to treat and prevent eclampsia is magnesium sulfate. Intravenous magnesium sulfate should be the initial drug administered to terminate seizures. A loading dose of 4 mg (15–20 min) and a maintenance dose of 1–2 g per hour as a continuous intravenous solution should be administered [54]. Management of severe hypertension (≥ 170/110 mm Hg) may include intravenous hydralazine or labetalol. Oral nifedipine may be an alternative treatment. Blood pressure should not be excessively decreased to prevent inadequate uteroplacental perfusion and fetal compromise. The goal is to maintain systolic blood pressure between 140 and 160 mm Hg and diastolic blood pressure around 90–110 mm Hg. Since a preterm delivery is required in women with eclampsia, a single course with betamethasone (12 mg IM q24h × 2 doses) may be administered for acceleration of fetal lung maturity. A systematic review and meta-analysis reported that antenatal steroids at ≥34 weeks’ gestation can reduce neonatal respiratory morbidity [55]

### 3.4. Thrombotic Microangiopathy

This complication, also called atypical hemolytic–uremic syndrome (HUS), is a medical emergency that may complicate the course of pregnancy or postpartum period. Thrombotic microangiopathy is characterized by microangiopathic hemolytic anemia, thrombocytopenia, and acute kidney injury. Its presentation is like preeclampsia. The differential diagnosis can be challenging [56]. Unfortunately, delays in appropriate diagnosis and treatment may be life-threatening. An international multidisciplinary working group proposed an approach that considers the timing of events in pregnancy or postpartum, coexisting symptoms, first-line laboratory workup, and probability-based assessment of possible causes of pregnancy-associated thrombotic microangiopathy. Deficiency of a disintegrin and metalloproteinase with thrombospondin-1 motifs (ADAMS13, a 13 member of the family) may diagnose a thrombotic thrombocytopenic purpura. Complement alternative pathway dysregulation indicates a diagnosis of atypical HUS [57]. Plasma exchanges and eculizumab are the first-line treatments for atypical HUS [58,59].

### 3.5. Kidney Dysfunction

Apart from pregnancy-related complications, other events may be responsible for graft function deterioration, including symptomatic or silent rejection, recurrence or de novo glomerular disease, and drug-related nephrotoxicity. Serum creatinine and proteinuria should be closely monitored in posttransplant pregnancy. Due to the physiologic alterations of kidney function induced by pregnancy, the baseline serum creatinine value should be that measured 4 weeks after conception, which is lower than that measured before pregnancy. A comprehensive analysis of various worldwide registers reported that the overall acute rejection rate during pregnancy among 822 kidney transplant recipients was 9.4% [5]. In some cases, rejection is not associated with signs and symptoms other than a mild increase in serum creatinine. In difficult cases, kidney biopsy is a reasonable option [60]. In cases of acute rejection, steroids and/or anti-thymocyte globulins (ATGs) are the preferred drugs. It should be noted that the trough blood levels of tacrolimus and, importantly, cyclosporine tend to decline at the second trimester [61]. Some investigators prefer to increase the doses of CNI, but the is no evidence that lower blood levels reflect the intracellular concentration of CNI [62].

Fetal complications are more frequent in women with poor graft function. A retrospective study estimated the effects of pregnancy renal function on pregnancy outcome. For women with serum creatinine < 1.4 m/dL, fetal growth restriction occurred in 25% of cases, preterm delivery in 30%, preeclampsia in 22%, and perinatal deaths in 1%. If serum creatinine was ≥2 mg/dL, fetal growth restriction occurred in 65% of cases, preterm delivery in >90%, preeclampsia in 60%, and perinatal deaths in 10% [63].

### 3.6. Diabetes Mellitus

During pregnancy, some women display metabolic abnormalities like those of people with type 2 diabetes mellitus, such as insulin resistance and reduced β-cell compensation for that resistance. This gestational diabetes may be defined as an increase in the fasting plasma glucose level of 6.9 mg/dL, an increase in the 1 h plasma glucose level of 30.9 mg/dL, and an increase in the 2 h plasma glucose level of 23.5 mg/dL [64]. In kidney transplant recipients, gestational diabetes develops in 16 % of pregnancies [65]. A systematic review and meta-analysis did not find a significant difference in the incidence of gestational diabetes between tacrolimus- and cyclosporine-treated transplant pregnancies [46]. Several studies in nontransplant women reported that gestational diabetes is associated with adverse pregnancy outcome [66,67]. All women with gestational diabetes mellitus should receive nutritional counseling, be instructed in self-monitoring of blood glucose, and should increase physical activity to moderate intensity levels if not contraindicated. The treatment goal is to maintain fasting blood glucose levels <95 mg per dL and 1 h postprandial <140 mg/dL. About 15–30% of patients who do not meet these goals should begin insulin therapy [68].

In summary, despite the risk of several complications, single-center and registry reports [69,70,71,72,73,74] and a meta-analysis [75] found that pregnancies in kidney transplant recipients with normal kidney function did not affect short-term and long-term patient and graft survival. However, the risk of graft dysfunction during pregnancy or in the postpartum period may be elevated in patients who became pregnant early after transplantation [14,15,16,17] and in those with elevated levels of serum creatinine [76,77]. A word of caution may concern the long-term impact on a patient who develops preeclampsia. A systematic review and meta-analysis in nontransplant women reported that preeclampsia significantly increases the risk of end-stage renal disease [78].

## 4. Immunosuppressive Drugs and Fetal Outcome

A major issue is represented by the potential teratogenic or toxic effects on the fetus (Table 2). The immunosuppressive agents more frequently used in kidney transplantation may be divided into three main categories: drugs that are relatively safe, new drugs with uncertain safety, and drugs with moderate to high fetal risk (Table 2).

### 4.1. Relatively Safe Drugs

Glucocorticoids easily cross the placenta [79] but are metabolized within the placenta by 11β-hydroxysteroid dehydrogenase-2 (11β-HSD2) which converts cortisol and synthetic glucocorticoids into inactive products [80,81]. A short course of poorly metabolized betamethasone can be used to prevent fetal respiratory distress in selected cases. Only excessive glucocorticoid administration can influence fetal growth [82,83,84] and results in dysfunction of the fetal hypothalamic–pituitary–adrenal axis [85]. This phenomenon, termed early life programming, may be associated in the long-term with impaired brain growth and increased susceptibility to cardiovascular disease. However, since only 3% of maternal cortisol is transferred to the fetal circulation [86], it is unlikely that moderate doses of exogenous glucocorticoids may interfere with the early life programming, unless there are alterations of 11β-HSD2 activities, as in the case of preeclampsia [87]. There has been concern about a possible increase in oral–facial clefts in newborns from mothers receiving glucocorticoids. This risk is 1.3 to 3.3 for every 1000 pregnancies exposed to glucocorticoids versus a birth population prevalence of 1 per 1000 [88]. Further studies confirmed that the risk of oral–facial cleft associated with prenatal exposure to glucocorticoids is minimal [89].

Calcineurin inhibitors. Reduced fetal weight, low birth weight and skeletal retardation, cesarean delivery, and hypertensive disorders of pregnancy have been frequently reported in pregnant transplanted women given CNIs [90]. However, transplant recipients are treated with many drugs, including other immunosuppressant agents, that may lead to confounding results. CNIs can cross the placenta [91]. The maternal–fetal transplacental passage can be influenced by the functional activity of P-glycoprotein [92]. Clinical studies reported that cyclosporine does not appear to be teratogenic [93,94]. The FDA classifies cyclosporine as category C, meaning that human risk cannot be ruled out. No lingering effects due to breast-feeding have been found in infants who were breast-fed while their mothers were taking cyclosporine. Tacrolimus crosses the placenta with exposure in utero being approximately 71% of maternal blood concentrations. The lower fetal blood concentrations are likely due to active efflux transport of tacrolimus from the fetus toward the mother by placental P-glycoprotein [95]. In newborns from transplant recipients, the risk of major malformations was low. CNIs can cause both reversible nephrotoxicity and hyperkalemia in the newborn. In summary, in pregnant women, the fetal outcomes are not particularly influenced by CNIs. Large reviews of pregnant transplanted women taking CNIs showed that the fetal outcomes of transplanted patients were comparable with those of nontransplanted patients with similar levels of kidney function impairment [96]. The relatively large number of premature births that has been reported by some series may be partially explained by an obstetric policy favoring earlier delivery [97]. Nonetheless, long-term effects in humans prenatally exposed to CNIs require further evaluation.

Azathioprine (AZA) is an orally absorbed prodrug, being a modification of 6-mercaptopurine, which is an analogue of the purine basis hypoxanthine. Based on animal studies, the FDA classified AZA in class C, as a drug at potential risk of teratogenic effects. The human placenta is a relative barrier to 6-mercaptopurine and together with the poor fetal metabolism of 6-mercaptopurine can explain the lack of proven teratogenicity in humans [98]. A systematic review showed that the risk of congenital anomalies in offspring, as well as the infertility risk, are similar than those found in the general population; however, there is a higher incidence of prematurity, of lower weight at birth, and an intrauterine delay of development in AZA-treated pregnant women [99]. Thus, the use of AZA in pregnancy appears to be relatively safe.

Mammalian target of rapamycin (mTOR) inhibitors. Sirolimus and its derivate everolimus are two immunosuppressive drugs with similar chemical structure that inhibit the proliferation of T cells by interfering with a serine–threonine kinase, called mTOR [100]. According to the FDA, sirolimus and everolimus are classified in category C. Animal reproduction studies have shown an adverse effect on the fetus. There are no adequate and well-controlled studies in humans, but potential benefits may warrant use of the drug in pregnant women despite potential risks. Episodic reports of successful pregnancy outcomes with exposure to sirolimus [101,102] or everolimus [103,104,105] have been given on pregnant kidney transplant recipients. 

Anti-thymocyte globulins (ATGs) are relatively safe medications with side effects mostly related to allergic reactions, nausea, vomiting, and diarrhea. There are no reports of fetal adverse events caused by ATG in humans. In an animal study, ATG caused decreased placental development in a murine model [106]. ATG has been successfully used to treat rejection in pregnant women [107].

Belatacept is a selective T cell costimulation blocker used to prevent rejection in organ transplantation. In animal studies, this drug, when given during organogenesis, was not teratogenic. Exposure to belatacept during pregnancy does not appear to be associated with an increased risk of birth defects Anecdotal cases of successful pregnancy and delivery with the use of belatacept-based immunosuppression have been reported [108,109].

### 4.2. Drugs with Uncertain Safety

Rituximab (RTX). Rituximab is a human/murine, chimeric anti-CD20 monoclonal antibody with established efficacy, and a favorable safety profile. RTX may cross the placenta and increase the risk of B cell depletion and other cytopenias in the neonate. According to the FDA, a teratogenic risk cannot be excluded for RTX (category C). Women may inadvertently become pregnant during or after RTX treatment. In a retrospective study, 153 women with malignancy or autoimmune disease were given RTX during pregnancy. Of them, 90 (59%) resulted in live births, and a high rate of miscarriages (22%) was attributed to concomitant therapy. Two congenital malformations were identified [110]. In a systematic review of 102 pregnancies, no major safety signal was observed with RTX use within 6 months of conception [111]. The long-term effects of in utero exposure to RTX are unknown. There is agreement that the administration of RTX to a pregnant woman should be discouraged unless the benefits outweigh the potential risk for the fetus [112,113]. 

Eculizumab is a monoclonal antibody directed against complement protein 5. It is used in kidney transplantation to prevent and treat atypical HUS and antibody-mediated rejection. There is a selective transport of unbound eculizumab across the human placenta. The levels observed in umbilical cord blood samples do not affect the concentrations of complement in newborns [114]. There is little experience with the use of eculizumab in pregnant women. Most data come from pregnancies in women with paroxysmal nocturnal hemoglobinuria. In 75 pregnancies, there were no maternal deaths and three fetal deaths (4%). Six miscarriages (8%) occurred during the first trimester [115]. Theoretically, eculizumab may cause terminal complement inhibition in the fetal circulation. However, in a study on two newborns from mothers treated with eculizumab during pregnancy, the antibody neither accumulated in fetal plasma nor impaired the complement function in the newborn [116]. The drug should be administered to pregnant women only if benefits may justify the potentially increased risk for the fetus.

### 4.3. Drugs with Fetal Toxicity

Mycophenolate salts. Use of mycophenolate drugs during pregnancy is associated with an increased risk of first trimester pregnancy loss and increased risk of congenital malformations [117,118,119]. The FDA classifies these drugs as category D., i.e., with positive evidence of human fetal risk. It is recommended that pregnancy be avoided by women taking MPA. Women being considered for treatment with MPA should always have a negative pregnancy test and should employ at least two methods of contraception during its use [120,121,122,123]. If pregnancy occurs, mycophenolate should be stopped as early as possible. The later mycophenolate is discontinued, the higher is the risk of complications. Little information is available on the long-term effects in infants born to mothers who had been exposed to mycophenolate.

Bortezomib is a reversible inhibitor of the 26S proteasome complex involved in degradation of ubiquitinated proteins. In kidney transplantation, bortezomib is used to prevent and treat antibody-mediated rejection. This drug has caused embryo–fetal lethality in animal studies at doses lower than the clinical dose. There are no controlled data in human pregnancy. Its use in pregnancy is not recommended. 

Cyclophosphamide is rarely used in kidney transplantation. Its teratogenesis is well demonstrated in animals. The drug is mutagenic and can cause urogenital malformations including unilateral renal agenesis [124]. The gene mutations induced by cyclophosphamide are dose-dependent and cumulative. The drug is considered as class D by the FDA. Miscarriages and pre-term deliveries have been reported in mothers taking cyclophosphamide. Cases of malformation have been described in newborns of mothers who received cyclophosphamide in the first months of pregnancy. The drug is contraindicated in pregnancy, particularly in the first trimester [125,126].

### 4.4. Vaginal or Cesarean Delivery

Cesarean section is frequently used to manage maternal issues, including poorly controlled diabetes, severe hypertension, and prolonged labor, or fetal disorders, such as malpresentation, non-reassuring heart rate, and growth restriction. However, a systematic review and meta-analysis reported that the complications related to Cesarean section in renal transplant recipients were 56.9%, higher than the 31.9% in the general US population [35]. Normal vaginal delivery is probably the safest method of delivery in patients with kidney transplantation, and Cesarean section should be performed only for strict medical or obstetrical indications. Careful preparation for Cesarean section in renal transplant recipients is imperative. Providers should be aware of the anatomy of a transplanted kidney and vascular anastomosis. In the event of surgery, care should be taken to avoid injury to the ureter of the kidney allograft, which may be anterior to the uterine artery [127]. A transabdominal ultrasound should be performed to localize the kidney prior to surgery. A clear understanding of the anatomic changes after kidney transplantation can help decrease the risk of injury [128]. 

## 5. Conclusions

Pregnancy is not contraindicated in transplant women, but it should be planned with an obstetrician and nephrologist. Ideally, the patient should not become pregnant 1-2 years after transplantation and should have normal blood pressure and kidney function. The maternal risks include hypertension, preeclampsia, thrombotic microangiopathy, graft dysfunction, gestational diabetes, and infection. The main fetal risks include prematurity, low birth weight, and abortion, particularly in patients with poor graft function. Renin–angiotensin system inhibitors and some immunosuppressive agents, such as mycophenolate, bortezomib, and cyclophosphamide, are teratogenic and should not be prescribed to pregnant women. If these recommendations are respected, pregnancy can be considered a safe option in kidney transplant women.

## Figures and Tables

**Table 1:** Main maternal adverse events during pregnancy after kidney transplantation.

Complication	Incidence	Consequences
Infection	Very common. Urinary tract infection (often asymptomatic) may affect most transplant women.	Urinary tract infections can increase the risk of preeclampsia. Pyelonephritis is an uncommon but severe complication. The risk of viral infection is elevated in case of early pregnancy. Infection may lead to graft dysfunction.
Hypertension	Many transplanted women are already hypertensive before pregnancy.	Hypertension can predispose to preeclampsia and is a main risk factor for cardiovascular disease, cerebrovascular disease, and graft dysfunction.
Preeclampsia	The incidence is higher in patients with poor graft function or hypertension.	Preeclampsia may result in damage to the kidneys, liver, lung, heart, or eyes, and may cause a stroke or other brain injury.
Gestational diabetes mellitus	About 16%	A maternal and neonate risk factor of adverse events during pregnancy.
Acute rejection	About 10%	Risk factor for graft dysfunction. Infection may develop after aggressive immunosuppressive treatment.

**Table 2:** Potential teratogenic effects of immunosuppressive drugs.

**Glucocorticoids (GC)**	GC can cross the placenta but are metabolized by 11 β-HSD2 to inactive products with the exception of dexamethasone and betamethasone. Fetal toxicity cannot be ruled out (Class C according to FDA).	Minimal risk of oral-facial cleft. It is unlikely that moderate doses of GC lead to fetal hypothalamic-pituitary-adrenal axis dysfunction or interfere with the early life programming, since 11β-hydroxysteroid dehydrogenase 2 is a major barrier against cortisol transfer to the fetus. Concern remains about the fetal toxicity when the mothers receive prolonged treatments with high-dose GC.
**Calcineurin inhibitors (CNI)**	CNI metabolites can be seen in the placenta. The CNI maternal–fetal transplacental passage is influenced by the activity of P-glycoprotein that pumps CNI out of the trophoblast cells of placenta and restricts its passage across the placental barrier. CNI are listed in Class C.	The relatively high number of premature births that has been reported may be partially explained by an obstetric policy favoring earlier delivery. Nonetheless, long-term effects in humans prenatally exposed to CNIs require further evaluation.
**Azathioprine (AZA)**	Placenta is a barrier to 6-mercaptopurine the main metabolite of AZA. This explains the lack of teratogenicity of AZA. The FDA classified AZA as a drug at potential risk of teratogenic effects (Class C) based on animal studies.	The risk of congenital anomalies in offspring are similar to those found in general population, but there is a higher incidence of prematurity and lower weight at birth in AZA-treated pregnant women. There is also an increased risk of materno-fetal infections, especially CMV infection. The use of AZA is considered safe.
**Mycophenolic acid (MPA)**	MPA during pregnancy is associated with an increased risk of congenital malformations. MPA is considered as class D (Evidence of risk to human fetus) by FDA.	Women being considered for treatment with MPA should always have a negative pregnancy test. There is an increased risk of pregnancy loss and congenital malformations with the use of MPA during pregnancy. If pregnancy occurs MPA should be stopped. The later MPA is discontinued the higher is the risk of complications.
**mTOR inhibitors (mTORi)**	Animal reproduction studies have shown an adverse effect on the fetus. There are not adequate and well-controlled studies in humans, but potential benefits may warrant use of the drug in pregnant women despite potential risks; mTORi are considered as class C by FDA.	The mTORi exposure during pregnancy does not appear to be associated with an increased risk or a pattern of birth defects.
**Rituximab (RTX)**	Data on RTX during pregnancy are scarce. RTX contains an IgG and can cross the placenta. RTX is considered in class C.	The administration of RTX to a pregnant woman is discouraged unless the benefits outweigh the potential risk for the fetus.
**Eculizumab**	There is a selective transport of unbound eculizumab across the placenta. However, the levels observed in umbilical cord blood samples do not affect the concentrations of complement in newborns.	The use of eculizumab to pregnant women with paroxysmal hemoglobinuria nocturnal is associated with a high rate of fetal survival and a low rate of maternal complications. Therefore, eculizumab may be regarded as safe in pregnancy.
**Belatacept**	There are not formal studies for the use of Belimumab in pregnant women.	Anecdotal cases of successful pregnancy with the use of belatacept in transplant women have been reported.

## Data Availability

Data sharing not applicable. No new data were created or analyzed in this study.
